# Effect of Multidirectional Forging on the Grain Structure and Mechanical Properties of the Al–Mg–Mn Alloy

**DOI:** 10.3390/ma11112166

**Published:** 2018-11-02

**Authors:** Mikhail S. Kishchik, Anastasia V. Mikhaylovskaya, Anton D. Kotov, Ahmed O. Mosleh, Waheed S. AbuShanab, Vladimir K. Portnoy

**Affiliations:** 1 Department of Physical Metallurgy of Non-Ferrous Metals, National University of Science and Technology “MISiS”, Leninsky Prospekt 4, 119049 Moscow, Russia; tanais61@bk.ru (M.S.K.); kotov@misis.ru (A.D.K.); mosleh@misis.ru (A.O.M.); portnoy@misis.ru (V.K.P.); 2 Mechanical Engineering Department, Shoubra Faculty of Engineering, Benha University, 108 Shoubra St., 11629 Cairo, Egypt; 3 Marine Engineering Department, Faculty of Maritime Studies and Marine Engineering, King Abdulaziz University, 21589 Jeddah, Saudi Arabia; wabushanab@kau.edu.sa

**Keywords:** aluminum alloy, isothermal multidirectional forging, grain refinement, recrystallization, mechanical properties

## Abstract

The effect of isothermal multidirectional forging (IMF) on the microstructure evolution of a conventional Al–Mg-based alloy was studied in the strain range of 1.5 to 6.0, and in the temperature range of 200 to 500 °C. A mean grain size in the near-surface layer decreased with increasing cumulative strain after IMF at 400 °C and 500 °C; the grain structure was inhomogeneous, and consisted of coarse and fine recrystallized grains. There was no evidence of recrystallization when the micro-shear bands were observed after IMF at 200 and 300 °C. Thermomechanical treatment, including IMF followed by 50% cold rolling and annealing at 450 °C for 30 min, produced a homogeneous equiaxed grain structure with a mean grain size of 5 µm. As a result, the fine-grained sheets exhibited a yield strength and an elongation to failure 30% higher than that of the sheets processed with simple thermomechanical treatment. The IMF technique can be successfully used to produce fine-grained materials with improved mechanical properties.

## 1. Introduction

Ultrafine grain (UFG) aluminum-based alloys have potential for use in industrial applications, due to their superior mechanical properties, such as strength-to-weight ratio, strength, ductility, and fatigue toughness [1,2,3,4,5]. An additional advantage of the UFG materials is their ability to be deformed into complex-shaped parts by superplastic forming (SPF) [6,7,8,9]. Severe plastic deformation (SPD) methods [10] are widely applied to refine grain structure [11,12,13,14]. These processing methods include equal-channel angular pressing (ECAP) [13,14,15,16,17,18], torsion under hydrostatic pressure [19,20,21,22], repetitive corrugation and straightening (RCS) [23], accumulative roll bonding [24], and multidirectional forging (MDF) [25,26,27,28,29,30]. MDF and isothermal multidirectional forging (IMF) [31,32] are the easiest, cheapest, and most promising industrial application SPD techniques [29,31]. The principle of these methods is based on cyclic rotations of the sample by 90 degrees, to change the strain axis [28,29,30,31,32,33]. The strain per pass varies in a range of 0.2 to 0.7 [31,32,33,34,35,36,37,38,39]. 

The IMF parameters, such as strain per pass, temperature, and strain rate, should be optimized to achieve a uniform UFG structure. Many researchers have studied the IMF effect on the properties of aluminum- and magnesium-based alloys, and their microstructure after IMF [33,37,39,40,41,42,43]. Multiaxial compression can withstand large strains ∑*e* > 28 [44,45,46]. IMF increases the strength properties significantly, and refines the grain size to ~1 µm [37,38,44] or even to ~0.5 µm at large strains in aluminum [45], and subgrain size to ~0.2 µm [47]. 

Several studies precisely analyzed the strain effect on microstructure evolution during IMF [32,37,38,39,48]. An ultrafine grain structure was found to form in IMF-proceeded Al-based alloys due to continuous dynamic recrystallization [14,36,38]. Microstructure inhomogeneity is the critical IMF problem, due to the inhomogeneity of the strain in different points of the sample at compression. Large cumulative strain during MDF increases structure homogeneity [14,38,39,49,50,51], and ∑*e* = 6–10 is typically required to form a fine and close-to-homogeneous grain structure. Recently published data [38] demonstrated the high efficiency of IMF for grain refinement of complex alloyed Sc-bearing 1570C aluminum alloy. The authors showed that total strain of 8.4 at 325 °C and 450 °C produced grain sizes of 1.2 µm and 2.5 µm, respectively. Notably, that alloy contained nanoscale precipitates of Al_3_(Sc,Zr) phase, significantly stabilizing the grain structure after various thermomechanical treatments [52,53,54] and severe plastic deformation [31,38,49,55] via the Zener pinning effect [56,57]. Jandaghi et al. [34] studied the microstructure evolution during IMF of AA5056 alloy. The authors observed significant strain inhomogeneity during IMF and a bimodal grain structure after IMF. Decreasing the IMF temperature results in finer grain structure formation, and IMF at cryogenic temperature (−196 °C) provided a homogeneous structure at a low strain of 2.4 [37]. Thus, published data confirm that the grain size and structure homogeneity are significantly affected by IMF temperature and cumulative strain. 

Despite the well-developed description of the IMF process and its effectiveness for grain refinement of different materials, there is no complete scientific understanding of the grain structure evolution during IMF of various commercial aluminum-based alloys. This study focuses on the analysis of strain-induced microstructure evolution after IMF of Al–Mg commercial alloy in a temperature range of 200 to 500 °C, and the comparison of the grain structure and properties of sheets processed with IMF or traditional hot rolling.

## 2. Materials and Methods

### 2.1. Materials and Processing

A commercial Al–Mg-based alloy (Russian grade 1565ch [58]) was studied. The chemical composition of the investigated alloy is shown in Table 1. The pure Al (99.7 wt %), pure Mg (99.5 wt %), pure Zn (99.5 wt %), and Al–10 wt %Cr, Al–10 wt %Mn master alloys were used to produce the samples. The master alloys were added to molten pure Al, and then pure Zn and Mg were added at the end of the melting process. The ingots were cast in laboratory conditions using a 100 mm × 40 mm × 20 mm water-cooled copper mold, and a casting cooling rate of approximately 15 °C/s. Melting was conducted in a Nabertherm S3 (Nabertherm GmbH, Lilienthal, Germany) electric furnace and graphite-fireclay crucibles, and the temperature before casting was 770 °C. As-cast samples were subjected to homogenization at 430 ± 2 °C for 5 h and 480 ± 2 °C for 3 h.

**Figure 1:**
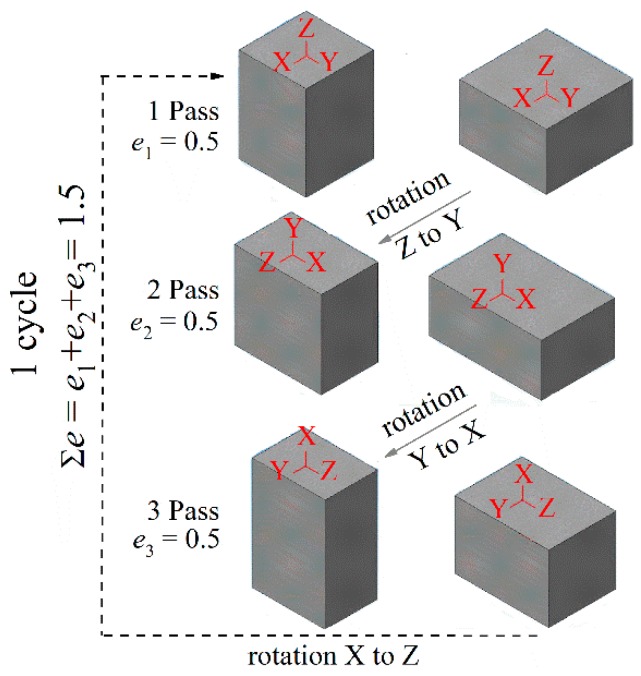
The scheme of the sample rotation during isothermal multidirectional forging (IMF).

The 14 mm × 10 mm × 10 mm samples for IMF were cut by electrical discharge machining. IMF was performed using a universal testing machine Walter Bay (Walter + Bai AG, Löhningen, Switzerland) equipped with a furnace (Walter + Bai AG, Löhningen, Switzerland) in isothermal conditions. IMF was performed at 200 °C (473 K), 300 °C (573 K), 400 °C (673 K), and 500 °C (773 K) at a strain rate of 6 × 10 s^−1^. Samples were maintained at the deformation temperature for 20 min before starting each compression. One IMF cycle included 3 forging passes (Figure 1) with a strain per pass of 0.5. Three steps were used to perform one IMF cycle with a total strain of 1.5, with the third pass returning the sample to its initial geometry. The total cumulative stress at IMF varied from 1.5 to 6.0. The compression procedures were performed in a die to limit the deformation in one direction and control the sample geometry. Graphite-based lubricant (Liqui Moly, Ulm, Germany) was used to reduce friction between the die elements and samples. 

To produce the sheets, IMF-processed samples were subjected to cold rolling (rolling mill V-3P, GMT, Saint-Petersburg, Russia) with a 50% reduction. The simple thermomechanical treatment without IMF was performed to produce the samples for comparison. Rolling at 200 or 400 °C with a reduction of 88% (*e* = 2.1) was used instead of IMF.

### 2.2. Microstructures Analyses

The microstructure was studied using an Axiovert 200 MMAT (Carl Zeiss, Oberkochen, Germany) optical light microscope (LM), using polarized light and a Tescan-VEGA3 (Tescan Brno s.r.o., Kohoutovice, Czech Republic) scanning electron microscope (SEM) equipped with an electron-backscatter diffraction (EBSD) HKL NordlysMax detector (Oxford Instruments plc, Abingdon, UK) and with an energy dispersive X-ray spectrometer (EDS) X-MAX80 (Oxford Instruments plc, Abingdon, UK). The EBSD analysis was performed with a step size of 0.4 μm and a scan area of 250 × 250 μm. In the analysis, high-angle grain boundaries (HAGBs) were defined as boundaries with misorientation angles (Ѳ) greater than 15°, and low-angle grain boundaries (LAGBs) were defined as boundaries with 2° < Ѳ < 15°. 

Samples for microstructure analysis were prepared by Struers LaboPol-5 (Struers APS, Ballerup, Denmark) machine by mechanical grinding and final polishing in a SiC suspension, and a subsequent polishing in chlorine–alcohol electrolyte at a voltage of 20 V. Anode oxidizing in 10% (HF in H_3_BO_4_) water solution was used to control the grain structure. The samples were analyzed in the near-surface layer, and in the midsections after 2 and 4 cycles. The mean grain size was determined by a random secant method for at least 300 measurements. The error bars were calculated using a standard deviation of the measured data and a confidence probability of 95%. 

### 2.3. Tensile Tests

The mechanical properties of the sheets were analyzed using a uniaxial tensile test on a Zwick Z250 (Zwick Roell Group, Ulm, Germany) test machine at room temperature. The samples with a gauge section size of *F*_0_ = 3 × 1.2 mm^2^ and length *L*_0_ = 10 mm (according to ISO 6892-1 international standard) were cut from the sheets annealed at 450 °C for 20 min.

## 3. Results

### 3.1. Microstructure after Homogenization Annealing

**Figure 2:**
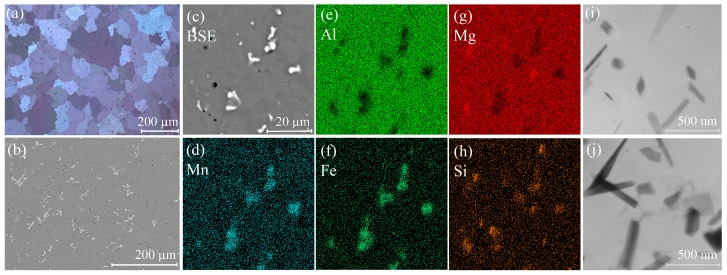
Microstructure after homogenization annealing (the initial state): (**a**) grain structure (light microscopy), (**b**,**c**) crystallization origin phases distribution, and (**d**–**h**) elements distribution maps of the (**c**) area (scanning-electron microscopy), and (**i**,**j**) transmission electron microscopy images of Mn-rich precipitates.

The initial microstructure after homogenization annealing is shown in the Figure 2. The as-homogenized sample exhibited a mean grain size of 89.7 ± 1.3 μm, and the grain size varied in the range of 20 to 200 µm. The alloy phase structure presented as an aluminum solid solution, and agglomerations of coarse crystallization origin inclusions on the periphery of aluminum dendrite cells. Two types of crystallization origin phases were observed. As shown in the SEM-EDS maps (Figure 2c,d,f–h), the light particles containing Mn, Si, and Fe most probably belong to the α-Al_12_(Mn,Fe)_3_Si_2_ phase, and dark gray inclusions rich in Mg and Si to the Mg_2_Si phase. The volume fraction of the light Fe-rich particles was ~2%.

Typical for AA5000 series alloys, secondary precipitates of platelet shape were observed in the as-homogenized state. The precipitates most probably belong to the Al_6_Mn phase [59,60,61,62,63]. The platelet thickness ranged from 20 to 210 nm, and length from 70 to 720 nm. 

### 3.2. Stress–Strain Behavior at IMF

**Figure 3:**
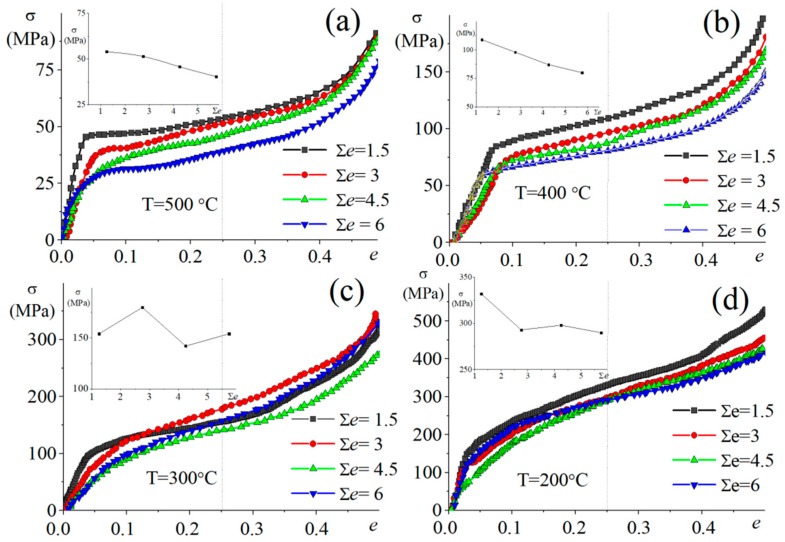
Stress–strain curves of 3, 6, 9, and 12 IMF passes at (**a**) 500 °C, (**b**) 400 °C, (**c**) 300 °C, and (**d**) 200 °C. The stress on the steady stage vs cumulative strain at each temperature is presented in the insets.

The stress–strain curves have similar shapes at different cumulative strains (Figure 3). The curves exhibited three stages: (1) primary stage at low strains up to ~0.05–0.08, (2) steady stage in a strain range of ~0.1 to ~0.35, and (3) stage with significant strain hardening at a strain above 0.35. Notably, stage 3, with a significant increase the stress, is the result of changing the deformation conditions at compression in a die, as well as friction effects.

The stress value during a steady stage monotonically decreased with increasing cumulative strain at 500 and 400 °C (inserts in Figure 3a,b), and non-monotonically depended on cumulative strain at lower temperatures of 300 and 200 °C. Thus, softening was observed at 500 and 400 °C, and the competition between softening and strain hardening effects was observed with increasing the cumulative strain at 300 and 200 °C.

### 3.3. Microstructure Evolution during IMF

Grain structure in the near-surface layer was studied after each pass of the first IMF cycle and after each next complete cycle (cumulative strain of 0.5, 1.0, 1.5, 3, 4.5 and 6.0). The sample surface (XZ plane in Figure 1) was ground and polished not more than 0.2–0.3 mm from the surface. The grain structure and the secondary phase distribution in a middle cross-section (half of the YXZ plane in Figure 1) were analyzed after two (∑*e* = 3) and four (∑*e* = 6) IMF cycles. 

#### 3.3.1. Grain Structure of the Near-Surface Layer

**Figure 4:**
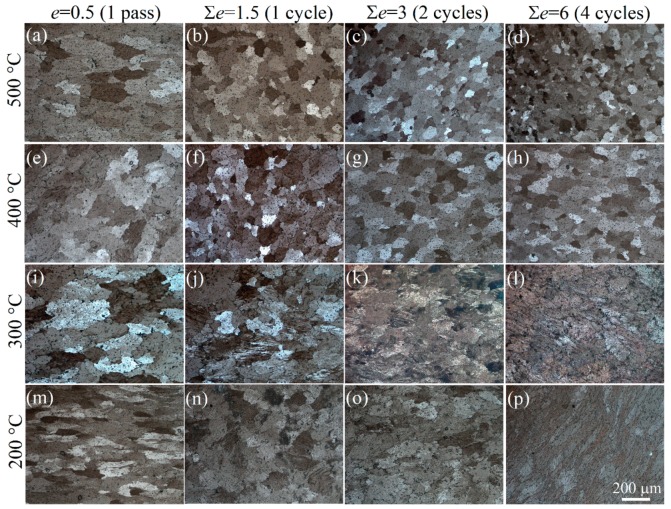
Microstructure of the samples in the near-surface layer after IMF for different strains (0.5–6.0) and temperatures (500–200 °C); detailed descriptions of temperatures and strains are provided in the figure.

Microstructures in near-surface layer, after subjection to various strains, are shown in Figure 4. Grains mostly retained an equiaxed shape close to the initial shape with a form factor of 0.85, and the grain shape insignificantly changed at both 500 and 400 °C (Figure 4a–h). 

**Figure 5:**
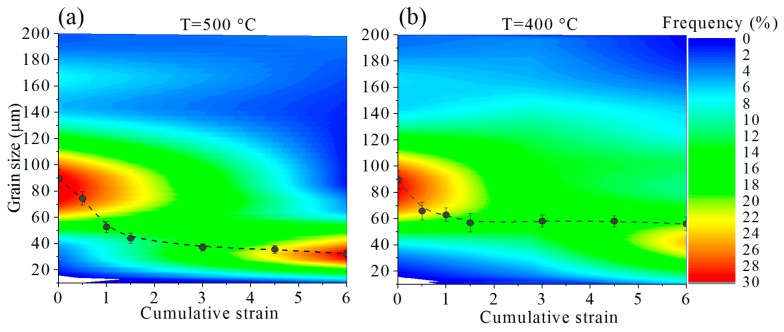
Grain size distribution after IMF process at (**a**) 500 °C and (**b**) 400 °C. Dashed lines show the mean grain size evolution.

The grain size distribution and mean grain size values, after various strains at 500 and 400 °C, are shown in Figure 5a,b respectively. After IMF at 500 °C (Figure 5a), the mean grain size in the subsurface layer decreased from 90 to 30 μm. A coarser grain structure on the surface was observed after IMF at 400 °C (Figure 5b), and mean grain size slightly decreased to ~60 μm at ∑*e* = 6.

The grain shape significantly changed at 300 and 200 °C: grains became elongated after the first compression with *e* = 0.5 with form factor of 0.56 (Figure 4i,m). Micro-shear bands [14] started to form inside several grains after ∑*e* = 1.5 (Figure 4j,n), and their amount significantly increased at ∑*e* = 3 (Figure 4k,o). Micro-shear bands formed inside most of the initial grains after ∑*e* = 6 (Figure 4l,p). Micro-shear bands formed in various directions due to the change in strain axis during IMF (Figure 4j–l,n–p).

#### 3.3.2. Grain Structure of Medium Cross-Section after ∑*e* = 3 and ∑*e* = 6

**Figure 6:**
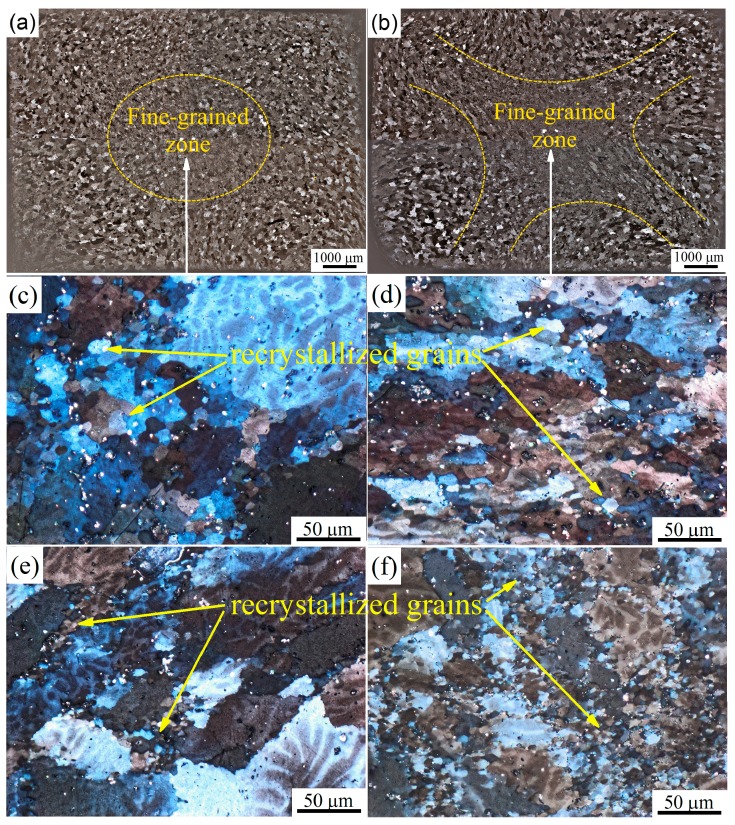
Structure of the median cross-section of the sample after (**a**,**c**) two (∑*e* = 3) and (**b**,**d**) four (∑*e* = 6) cycles at (**a**–**d**) 500 °C and (**e**,**f**) 400 °C.

Figure 6a,b shows the grain structure in a median cross-section of the IMF-processed samples after ∑*e* = 3 and ∑*e* = 6 at 500 °C. The grain structure in a sample volume was inhomogeneous: the grain size decreased from the surface to the center of the sample (Figure 6a). The strain increase from three to six helped to widen the fine-grained zone toward the periphery of the sample (Figure 6b). A similar grain distribution in a cross-section was observed at 400 °C.

Micrographs at higher magnification of the samples subjected to IMF at 500 °C (Figure 6c,d) and 400 °C (Figure 6e,f) showed additional inhomogeneity: coarse grains of 50–100 µm were surrounded by fine grains of 3–10 µm. The proportion of fine grains increased with increasing cumulative strain from three to six at both temperatures. Conversely, the mean size of the fine grains did not depend on strain. The mean grain size after IMF at 500 °C was 10.3 ± 0.5 μm and 10.2 ± 0.4 μm after ∑*e* = 3 (two cycles) and ∑*e* = 6 (four cycles), respectively. IMF at 400 °C produced a finer mean grain size in the fine-grained zone: 5.5 ± 0.5 μm and 5.3 ± 0.4 μm after ∑*e* = 3 and ∑*e* = 6, respectively. 

**Figure 7:**
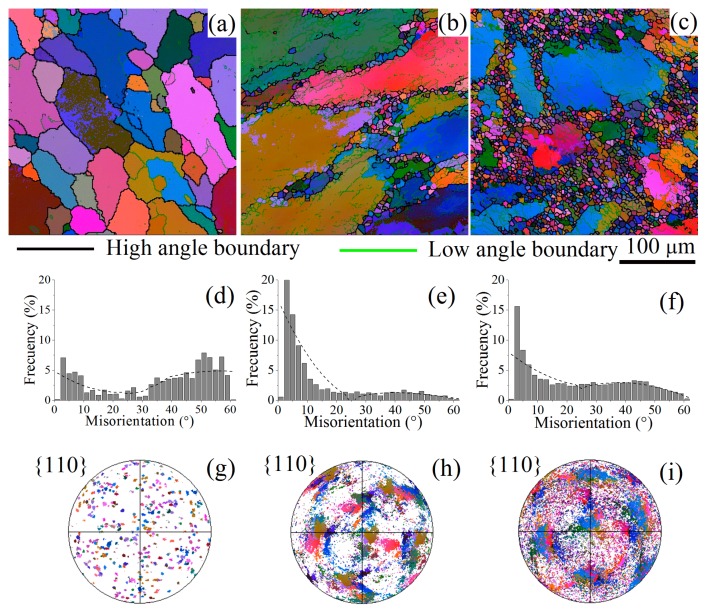
Grain distribution maps (**a**–**c**), boundary misorientation angle distributions (**d**–**f**), and the pole figures (**g**–**i**): as-homogenized (**a**,**d**,**g**); after two cycles (**b**,**e**,**h**); and after four cycles of forging at 400 °C (**c**,**f**,**i**).

The results of the EBSD study of the as-homogenized sample and the sample processed at 400 °C are shown in Figure 7 and Table 2. A coarse-grained structure with several low-angle boundaries (10%) was observed after homogenization. A strong texture formed, the fraction of low-angle grain boundaries with misorientation angles ≤15° increased to 50% after ∑*e* = 3, and fine equiaxed grains formed mainly near the initial grain boundaries.

Increasing the strain to ∑*e* = 6 resulted in the formation of many new fine grains with high-angle grain boundary misorientation (>15°), and corresponding decreasing fraction of low-angle grain boundaries and some texture removal. Thus, the EBSD analysis confirmed that the inhomogeneity of the grain structure and the fine recrystallized grains fraction increased with increasing cumulative strain.

**Figure 8:**
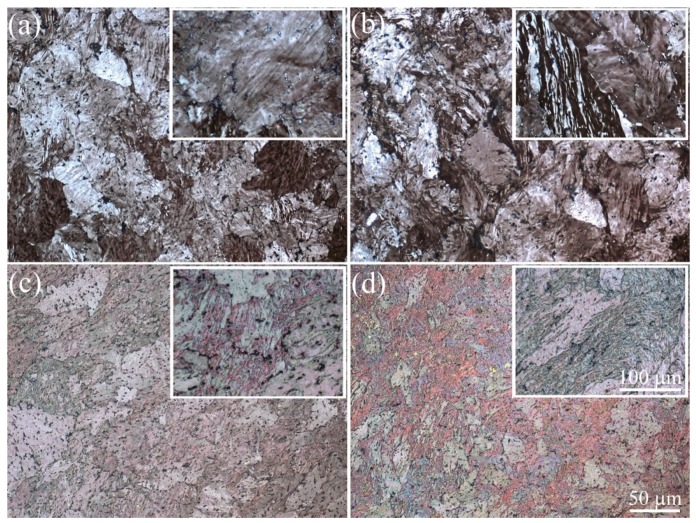
Structure of the median cross-section of the sample after (**a**,**c**) two (∑*e* = 3) and (**b**,**d**) four (∑*e* = 6) cycles at (**a**,**b**) 300 °C and (**b**,**d**) 200 °C.

Many micro-shear bands and fragments with weak contrast in polarized light inside each as-cast grain were observed in the middle cross-section after IMF at lower temperatures of 300 and 200 °C (Figure 8). The micro-shear bands crossed each other and formed tangles as a result of increasing strain. The areas free of micro-shear bands grain (light zones in Figure 8) were also observed after ∑*e* = 6.

#### 3.3.3. Evolution of the Secondary Phases

**Figure 9:**
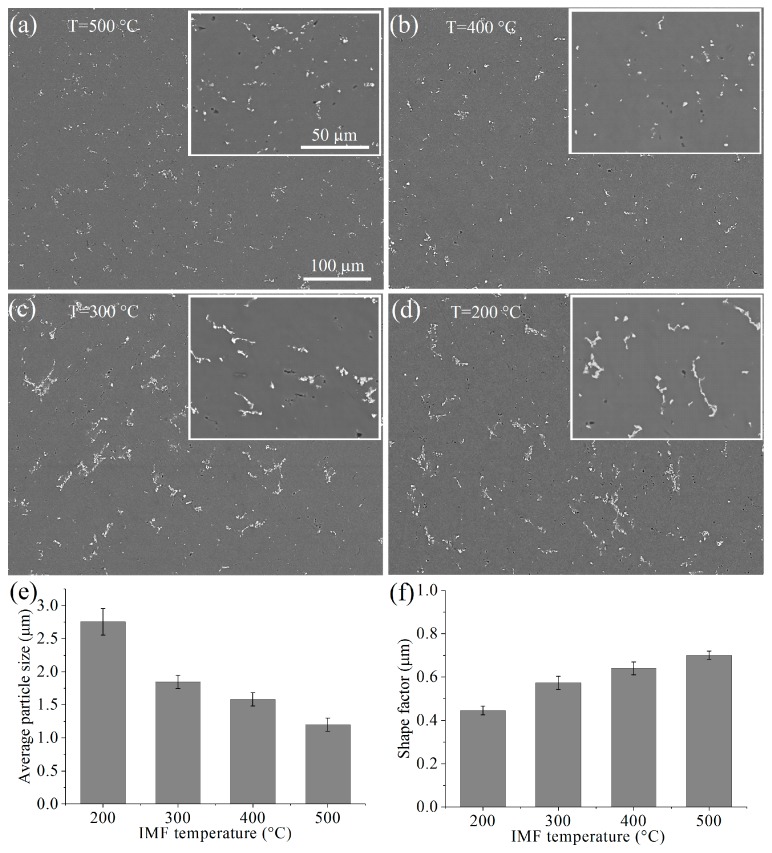
Microstructures of the samples after four cycles (∑*e* = 6) IMF at (**a**) 500, (**b**) 400, (**c**) 300, and (**d**) 200 °C, and (**e**) average particle size and (**f**) shape factor dependence on IMF temperature (scanning electron microscope).

As mentioned above (Figure 2), the alloy microstructure after homogenization occurred as an aluminum solid solution and agglomerations of crystallization origin inclusions. The mean particle size decreased from 2.75 µm to 1.75 µm (Figure 9e), and the form factor increased from 0.45 to 0.7 (Figure 9f) with increasing IMF temperature from 200 to 500 °C. Thus, IMF at 400 and 500 °C with ∑*e* = 6 refined the crystallization origin inclusions, and increased their homogenous distribution in the aluminum matrix (Figure 9a,b,e,f). Particle agglomerations were unobserved after IMF at 400 and 500 °C. The IMF less significantly influenced on the inclusion size and their distribution at lower temperatures of 200 and 300 °C (Figure 9c–f).

### 3.4. Grain Structure and Mechanical Properties of the Sheets

The samples were subjected to thermomechanical treatment using deformation regimes with IMF at 400 °C and 200 °C, and without IMF (Table 3). The first mode (A1) is typical for industrial processing.

**Figure 10:**
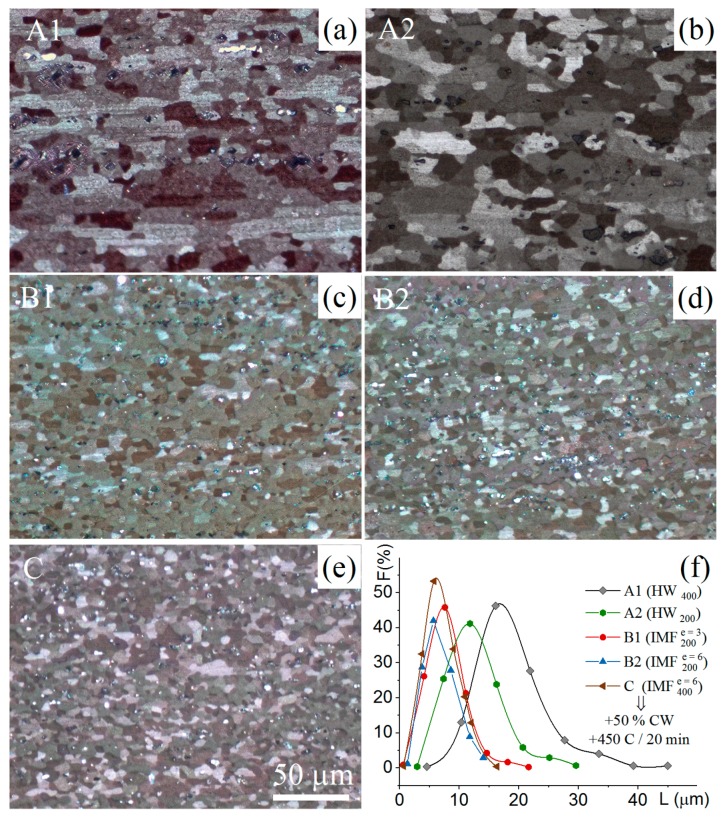
The microstructures of cold-rolled sheets after annealing at 450 °С for 20 min for (**a**) A1, (**b**) A2, (**c**) B1, (**d**) B2, and (**e**) C treatment modes and (**f**) frequency (*F*) vs grain size (*L*) for studied treatments modes.

The microstructures of cold-rolled sheets, after annealing at 450 °С for 30 minutes, are presented in Figure 10a–e. Applying IMF (modes B (Figure 10c,d) and C (Figure 10e)) instead of hot or warm rolling (modes A1 (Figure 10a) and A2 (Figure 10b)) produced a finer grain structure: the fraction of fine grains increased and the mean grain size decreased from 10–15 to 5 µm (Figure 10f and Table 4). Notably, the grain structure in the sheets processed with IMF was homogeneous, and grains had an equiaxed shape with form factor of ~0.9.

The ultimate tensile strength does not depend on the sheet processing type. Finer grain size provided higher values of yield strength and elongation to failure for the sheets subjected to thermomechanical treatment, including the IMF. 

## 4. Discussion

The microstructure evolution was affected significantly by IMF temperature and strain. All testing temperatures were above the 0.5T_melt_, where the diffusion process, recovery, and related phenomena significantly impact the deformation process, dislocation and grain structure evolution. IMF at 200 °C and 300 °C forms micro-shear bands [14,38,64], and no new recrystallized grains were observed. Micro-shear bands formed on the surface and in a volume of the deformed samples typical for IMF at 200–300 °C in the studied material. The deformation bands or micro-shear bands consist of fragments having a low-angle grain boundary misorientation [14,65]. The new fine equiaxed grains were not observed, and the grains elongated and maintained their initial mean size. Thus, the microstructure study suggested only dynamic recovery during IMF and subsequent annealing at 200 to 300 °C. 

Fine grain formation and decreasing stress value with increasing cumulative strain indicate the recrystallization phenomena during the IMF process at 400 °C and 500 °C. The new recrystallized grains were mostly observed on the boundaries of coarser grains. The same effect was found in the other alloys [14,32,38]. This finding is a result of the high inhomogeneity of deformation: a high fraction of strain-induced low-angle grain boundaries formed on the periphery of the initial as-cast grains. A dendrite pattern was observed in several coarse grains, which confirms their as-cast nature, even after large cumulative strains. Several grains had no inner dendrite pattern. Thus, we cannot exclude the possibility of coarser grains formation in a sample volume due to dynamic recrystallization and growth of the grains forming during previous cycles of deformation and subsequent heating and soaking at IMF temperature. 

Crystallization origin particles, that were refined to 1.2–1.6 µm, became more compact and homogeneously distributed in the aluminum matrix after the IMF at 400 and 500 °C The intensive fragmentation and spheroidization of the secondary particles were the result of both elevated temperature and strain-induced effects. It is important to note that both the recrystallization and evolution of the crystallization origin particles distribution were observed simultaneously. Thus, coarse particles can increase the rate of dynamic recrystallization during high temperature IMF and static recrystallization during annealing before the start of the next IMF cycle via a particle-stimulated nucleation (PSN) mechanism [66,67,68]. The PSN effect seems reasonable, due to the large cumulative strain during IMF. In addition, grain size analysis found high grain size stability in a fine-grained area. The fine secondary Mn-bearing precipitates should inhibit grain growth during hot deformation and heating at IMF temperature via the Zener pinning mechanism [67,69]. Thus, the grain structure after IMF was affected by both coarse and fine particles contained in the studied alloy. 

IMF was accompanied by significant increases in the fraction of low-angle grain boundaries. Therefore, continuous dynamic recrystallization [67] is suggested as the main recrystallization mechanism, in agreement with previous studies [14,34,38,68,70,71]. According to Humphreys [67], continuous dynamic recrystallization occurs due to the transformation of strain-induced low-angle grain boundaries into high-angle grain boundaries. A similar mechanism can occur during annealing of deformed material. Both dynamic/post-dynamic recrystallization [14,72], as a result of deformation, and static recrystallization during post-deformation annealing are possible in the studied alloy in a temperature range of 400 to 500 °C. It is suggested that static recrystallization occurred when samples were maintained at deformation temperature before the start of compression. The fine recrystallized grains mostly exhibited an equiaxed shape after compression, which suggested their dynamic recrystallization nature. 

The fine-grained zone increased with increasing cumulative strain, but IMF with strain of ∑*e* = 6 in a studied temperature range did not produce a homogeneous grain structure in the studied alloy. Similar inhomogeneity was observed in other Al- and Mg-based alloys, due to strain inhomogeneity at compression [19,26,31,34,35,39]. Despite the effect of grain inhomogeneity, applying IMF instead of hot or warm rolling helped to form a fine and homogenous grain structure in cold-rolled and subsequently recrystallized sheets. The final grain size decreased from 15 to 5 µm, thereby increasing both yield stress due to Hall–Petch strengthening, and elongation to failure of ~30%, compared to sheets processed with hot rolling. Comparatively large strain at both 200 and 400 °C can intensively refine the secondary precipitates (dispersoids) that were observed in prior studies [73,74,75,76,77,78,79,80]. The fine grain structure observed after thermomechanical treatment, including IMF, can be the result of both the effect of stimulation nucleation by grain boundaries at recrystallization and the stronger Zener drag force. 

In summary, IMF, even with low cumulative strain **∑***e* = 3, can be successfully used instead of hot rolling to produce fine-grained sheets with improved mechanical properties. Thus, IMF is a very promising SPD technique to produce good quality industrial products.

## 5. Conclusions

Microstructural evolution in commercial Al–Mg-based alloy, during isothermal multidirectional forging (IMF) in a temperature range of 200 °C to 500 °C and a strain range of 1.5 to 6, was investigated. Low-angle boundaries formed during IMF, with subsequent recrystallization at 400–500 °C with the formation of fine (3–10 µm) equiaxed grains at large strains. The mechanism of continuous dynamic recrystallization is suggested as the IMF grain refinement mechanism at temperatures of 400 and 500 °C. Elongation of the initial grains and intensive formation of micro-shear bands were observed at lower temperatures of 200 and 300 °C. The increased IMF temperature, from 200 to 500 °C, enabled the significant refinement of the crystallization origin inclusions and their homogeneous distribution in the aluminum matrix. 

Isothermal multidirectional forging at both 200 and 400 °C, with cumulative strain of three to six, helped to refine the grain structure after sheet processing. The mean grain size decreased from 15 µm for conventional thermomechanical treatment, including rolling, to 5 µm for thermomechanical treatment, including isothermal multidirectional forging. As a result of the finer grain structure, the yield strength and elongation to failure of the sheets processed with IMF were 30% higher compared with pre-rolled materials.

## Figures and Tables

**Table 1:** Chemical composition of the studied alloy.

**Element**	Mg	Mn	Zn	Fe	Si	Zr	Cr	Al
**Concentration (wt %)**	5.7	0.8	0.7	<0.2	<0.1	0.1	0.1	Base

**Table 2:** The EBSD data of IMF-proceeded samples at 400 °C.

**Parameter**	*e* = 0.5 (1 pass)	Σ*e* = 3 (2 cycles)	Σ*e* = 6 (4 cycles)
**Grain size (μm)**	45.1	5.5/fine grained area	5.3/fine grained area
**LAGBs volume fraction (%)**	24.2	73.5	44.0

Definitions: EBSD—electron backscatter diffraction; IMF—isothermal multidirectional forging; e—strain; and LAGBs—low-angle grain boundaries.

**Table 3:** Thermomechanical treatment types.

Modes A1 and A2	Modes B1 and B2	Mode C
Rolling at 400 °C (A1) or at 200 °C (A2), ∑*e* = 2.1; Cold rolling, *e* = 0.69	IMF at 200 °C, ∑*e* = 3 (B1), ∑e = 6 (B2); Cold rolling, *e* = 0.69	IMF at 400 °C, ∑*e* = 6; Cold rolling, *e* = 0.69

**Table 4:** Mechanical properties of sheets.

Scheme of Thermomechanical Treatment	Mean Grain Size (µm)	Mechanical Property		
		Yield Strength (MPa)	Ultimate Tensile Strength (MPa)	Elongation (%)
A1	15.6 ± 0.8	150 ± 3	360 ± 3	21 ± 1
A2	10.4 ± 0.5	160 ± 2	362 ± 3	22 ± 1
B1	7.1 ± 0.3	205 ± 1	362 ± 1	27 ± 1
B2	5.1 ± 0.2	205 ± 2	368 ± 4	27 ± 1
C	5.0 ± 0.3	200 ± 2	363 ± 4	27 ± 1

## References

[1] Nikulin I., Kipelova A., Malopheyev S., Kaibyshev R. (2012). Effect of second phase particles on grain refinement during equal-channel angular pressing of an Al-Mg-Mn alloy. Acta Mater..

[2] Robson J.D. (2004). Microstructural evolution in aluminium alloy 7050 during processing. Mater. Sci. Eng. A.

[3] Shabani M.J., Emamy M., Nemati N. (2011). Effect of grain refinement on the microstructure and tensile properties of thin 319 Al castings. Mater. Des..

[4] Grains U., Zhemchuzhnikova D., Lebyodkin M. (2017). Peculiar Spatiotemporal Behavior of Unstable Plastic Flow in an AlMgMnScZr Alloy with Coarse and Ultrafine Grains. Metals.

[5] Murashkin M., Medvedev A., Kazykhanov V., Krokhin A., Raab G., Enikeev N., Valiev R.Z. (2015). Enhanced Mechanical Properties and Electrical Conductivity in Ultrafine-Grained Al 6101 Alloy. Processed via ECAP-Conform. Metals.

[6] Barnes A.J. (2001). Industrial Applications of Superplastic Forming: Trends and Prospects. Mater. Sci. Forum.

[7] Grimes R., Dashwood R.J., Flower H.M. (2001). High Strain Rate Superplastic Aluminium Alloys: The Way Forward. Mater. Sci. Forum.

[8] Langdon T.G. (2013). Achieving superplasticity in ultrafine-grained metals. Mech. Mater..

[9] Kawasaki M., Langdon T.G. (2011). Developing superplasticity and a deformation mechanism map for the Zn–Al eutectoid alloy processed by high-pressure torsion. Mater. Sci. Eng. A.

[10] Segal V.M. (1995). Materials processing by simple shear. Mater. Sci. Eng. A.

[11] Markushev M.V., Avtokratova E.V., Sitdikov O.S. (2017). Effect of the initial state on nanostructuring and strengthening of middle- and high-strength age-hardenable aluminum alloys under severe plastic deformation (Review). Lett. Mater..

[12] Kawasaki M., Ahn B., Kumar P., Jang J., Langdon T.G. (2017). Nano- and Micro-Mechanical Properties of Ultrafine-Grained Materials Processed by Severe Plastic Deformation Techniques. Adv. Eng. Mater..

[13] Langdon T.G. (2013). Twenty-five years of ultrafine-grained materials: Achieving exceptional properties through grain refinement. Acta Mater..

[14] Sakai T., Belyakov A., Kaibyshev R., Miura H., Jonas J.J. (2014). Dynamic and post-dynamic recrystallization under hot, cold and severe plastic deformation conditions. Prog. Mater. Sci..

[15] Ning J.L., Jiang D.M. (2007). Influence of Zr addition on the microstructure evolution and thermal stability of Al-Mg-Mn alloy processed by ECAP at elevated temperature. Mater. Sci. Eng. A.

[16] Ning J.L., Jiang D.M., Fan X.G., Lai Z.H., Meng Q.C., Wang D.L. (2008). Mechanical properties and microstructure of Al-Mg-Mn-Zr alloy processed by equal channel angular pressing at elevated temperature. Mater. Charact..

[17] Zhao S., Meng C., Mao F., Hu W., Gottstein G. (2014). Influence of severe plastic deformation on dynamic strain aging of ultrafine grained Al-Mg alloys. Acta Mater..

[18] Fritsch S., Wagner M.F. (2018). On the Effect of Natural Aging Prior to Low Temperature ECAP of a High-Strength Aluminum Alloy. Metals.

[19] Xu X., Zhang Q., Hu N., Huang Y., Langdon T.G. (2013). Using an Al–Cu binary alloy to compare processing by multi-axial compression and high-pressure torsion. Mater. Sci. Eng. A.

[20] Xu C., Horita Z., Langdon T.G. (2008). The evolution of homogeneity in an aluminum alloy processed using high-pressure torsion. Acta Mater..

[21] Zhilyaev A.P., Langdon T.G. (2008). Using high-pressure torsion for metal processing: Fundamentals and applications. Prog. Mater. Sci..

[22] Murashkin M., Sabirov I., Prosvirnin D., Ovid I., Terentiev V., Valiev R., Dobatkin S. (2015). Fatigue Behavior of an Ultrafine-Grained Al-Mg-Si Alloy Processed by High-Pressure Torsion. Metals.

[23] Thangapandian N., Balasivanandha S.P., Padmanabhan K.A. (2016). Effects of die profile on grain refinement in Al-Mg alloy processed by repetitive corrugation and straightening. Mater. Sci. Eng. A.

[24] Daneshvar F., Reihanian M., Gheisari K. (2016). Al-based magnetic composites produced by accumulative roll bonding (ARB). Mater. Sci. Eng. B.

[25] Kavosi J., Saei M., Kazeminezhad M., Dodangeh A. (2014). Modeling of dislocation density and strength on rheoforged A356 alloy during multi-directional forging. Comput. Mater. Sci..

[26] Huang H., Zhang J. (2016). Microstructure and Mechanical properties of AZ31 magnesium alloy processed by multi-directional forging at different temperatures. Mater. Sci. Eng. A.

[27] Miura H., Nakao Y., Sakai T. (2007). Enhanced Grain Refinement by Mechanical Twinning in a Bulk Cu-30 mass%Zn during Multi-Directional Forging. Mater. Trans..

[28] Nakao Y., Miura H. (2011). Nano-grain evolution in austenitic stainless steel during multi-directional forging. Mater. Sci. Eng. A.

[29] Ghosh A.K., Huang W. (2000). Severe deformation based process for grain subdivision and resulting microstructures. Investigations and Applications of Severe Plastic Deformation.

[30] Belyakov A., Sakai T., Miura H. (2001). Microstructure and deformation behaviour of submicrocrystalline 304 stainless steel produced by severe plastic deformation. Mater. Sci. Eng. A.

[31] Sitdikov O.S., Avtokratova E.V., Mukhametdinova O.E., Garipova R.N., Markushev M.V. (2017). Effect of the Size of Al_3_(Sc,Zr) Precipitates on the Structure of Multi-Directionally Isothermally Forged Al-Mg-Sc-Zr Alloy. Phys. Met. Metallogr..

[32] Sitdikov O., Sakai T., Miura H., Hama C. (2009). Temperature effect on fine-grained structure formation in high-strength Al alloy 7475 during hot severe deformation. Mater. Sci. Eng. A.

[33] Dziubińskaa A., Gontarz A., Horzelska K., Pieśko P. (2015). The microstructure and mechanical properties of AZ31 magnesium alloy aircraft brackets produced by a new forging technology. Procedia Manuf..

[34] Jandaghi M.R., Pouraliakbar H., Gharah Shiran M.K., Khalajd G., Shirazie M. (2016). On the effect of non-isothermal annealing and multi-directional forging on the microstructural evolutions and correlated mechanical and electrical characteristics of hot-deformed Al-Mg alloy. Mater. Sci. Eng. A.

[35] Asadi S., Kazeminezhad M. (2017). Multi Directional Forging of 2024 Al Alloy After Different Heat Treatments: Microstructural and Mechanical Behavior. Trans. Indian Inst. Met..

[36] Moghanaki K.S., Kazeminezhad M. (2017). Effects of non-isothermal annealing on microstructure and mechanical properties of severely deformed 2024 aluminum alloy. Trans. Nonferrous Met. Soc. China.

[37] Rao P.N., Singh D., Jayaganthan R. (2014). Mechanical properties and microstructural evolution of Al 6061 alloy processed by multidirectional forging at liquid nitrogen temperature. Mater. Des..

[38] Sitdikov O., Garipova R., Avtokratova E., Mukhametdinova O., Markushev M. (2018). Effect of temperature of isothermal multidirectional forging on microstructure development in the Al-Mg alloy with nano-size aluminides of Sc and Zr. J. Alloys Compd..

[39] Armstrong P.E., Hockett J.E., Sherby O.D. (1982). Large Deformation of 1100 Aluminum at 300 K. J. Mech. Phys. Solids.

[40] Li J., Liu J., Cui Z. (2015). Microstructures and mechanical properties of AZ61 magnesium alloy after isothermal multidirectional forging with increasing rate. Mater. Sci. Eng. A.

[41] Miura H., Yu G., Yang X. (2011). Multi-directional forging of AZ61Mg alloy under decreasing temperature conditions and improvement of its mechanical properties. Mater. Sci. Eng. A.

[42] Xing J., Soda H., Yang X., Miura H., Sakai T. (2005). Ultra-Fine Grain Development in an AZ31 Magnesium Alloy during Multi-Directional Forging under Decreasing Temperature Conditions. Mater. Trans..

[43] Zhu Q., Li L., Ban C., Zhao Z., Zuo Y., Cui J. (2014). Structure uniformity and limits of grain refinement of high purity aluminum during multi-directional forging process at room temperature. Trans. Nonferrous Met. Soc. China.

[44] Łyszkowski R., Czujko T., Varin R.A. (2017). Multi-axial forging of Fe_3_Al-base intermetallic alloy and its mechanical properties. J. Mater. Sci..

[45] Bereczki P., Szombathely V., Krallics G. (2014). Production of ultrafine grained aluminum by cyclic severe plastic deformation at ambient temperature. IOP Conf. Ser. Mater. Sci. Eng..

[46] Padap A.K., Chaudhari G.P., Nath S.K., Pancholi V. (2009). Ultrafine-grained steel fabricated using warm multiaxial forging: Microstructure and mechanical properties. Mater. Sci. Eng. A.

[47] Petryk H., Stupkiewicz S., Kuziak R. (2008). Grain refinement and strain hardening in IF steel during multi-axis compression: Experiment and modelling. J. Mater. Process. Technol..

[48] Moghanaki K.S., Kazeminezhad M., Loge R. (2017). Effect of concurrent precipitation on the texture evolution during continuous heating of multi directionally forged solution treated Al-Cu-Mg alloy. Mater. Charact..

[49] Ringeva S., Piot D., Desrayaud C., Driver J.H. (2006). Texture and microtexture development in an Al–3Mg–Sc(Zr) alloy deformed by triaxial forging. Acta Mater..

[50] Maruff H., Rao P.N., Dharmendra S., Jayaganthan R., Singh S. (2014). Comparative Study of Microstructure and Mechanical Properties of Al 6063 alloy Processed by Multi Axial Forging at 77K and Cryorolling. Procedia Eng..

[51] Nakao Y., Miura H., Sakai T. (2007). Microstructural evolution and recrystallization behavior in copper multi-directionally forged at 77 K. Adv. Mater. Res..

[52] Mikhaylovskaya A.V., Yakovtseva O.A., Cheverikin V.V., Kotov A.D., Portnoy V.K. (2016). Superplastic behaviour of Al-Mg-Zn-Zr-Sc-based alloys at high strain rates. Mater. Sci. Eng. A.

[53] Kotov A.D., Mikhaylovskaya A.V., Kishchik M.S., Tsarkov A.A., Aksenov S.A., Portnoy V.K. (2016). Superplasticity of high-strength Al-based alloys produced by thermomechanical treatment. J. Alloys Compd..

[54] Kishchik A.A., Mikhaylovskaya A.V., Kotov A.D., Rofman O.V., Portnoy V.K. (2018). Al-Mg-Fe-Ni based alloy for high strain rate superplastic forming. Mater. Sci. Eng. A.

[55] Buranova Y., Kulitskiy V., Peterlechner M., Mogucheva A., Kaibyshev R., Divinski S.V., Wilde G. (2017). Al_3_(Sc,Zr)-based precipitates in Al–Mg alloy: Effect of severe deformation. Acta Mater..

[56] Smith C.S. (1948). Grains, Phases, and Interfaces—An Interpretation of Microstructure. Metall. Mater. Trans. A.

[57] Manohar P.A., Ferry M., Chandra T. (1998). Five decades of the Zener equation. ISIJ Int..

[58] Kishchik M.S., Mikhailovskaya A.V., Levchenko V.S., Kotov A.D., Drits A.M., Portnoy V.K. (2017). Formation of Fine-Grained Structure and Superplasticity in Commercial Aluminum Alloy 1565ch. Met. Sci. Heat Treat..

[59] Engler O., Miller-Jupp S. (2016). Control of second-phase particles in the Al-Mg-Mn alloy AA 5083. J. Alloys Compd..

[60] Lucadamo G., Yang N.Y.C., SanMarchi C., Lavernia E.J. (2006). Microstructure characterization in cryomilled Al 5083. Mater. Sci. Eng. A.

[61] Engler O., Kuhnke K., Westphal K., Hasenclever J. (2018). Impact of chromium on the microchemistry evolution during solidification and homogenization of the Al-Mg alloy AA 5052. J. Alloys Compd..

[62] Engler O., Liu Z., Kuhnke K. (2013). Impact of homogenization on particles in the Al–Mg–Mn alloy AA 5454–Experiment and simulation. J. Alloy. Compd..

[63] Yi G., Sun B., Poplawsky J.D., Zhu Y., Free M.L. (2018). Investigation of pre-existing particles in Al 5083 alloys. J. Alloys Compd..

[64] Jandaghi M.R., Pouraliakbar H. (2017). Study on the Effect of Post-Annealing on the Microstructural Evolutions and Mechanical Properties of Rolled CGPed Aluminum-Manganese-Silicon alloy. Mater. Sci. Eng. A.

[65] Sitdikov O., Sakai T., Goloborodko A., Miura H. (2004). Grain fragmentation in a coarse-grained 7475 Al alloy during hot deformation. Scr. Mater..

[66] Nes E. (2001). Primary Recrystallization in Two-phase Alloys. Encycl. Mater. Sci. Technol..

[67] Humphreys J., Rohrer G.S., Rollett A. (2004). Recrystallization and Related Annealing Phenomena.

[68] Churyumov A.Y., Mikhaylovskaya A.V., Bazlov A.I., Tsarkov A.A., Kotov A.D., Aksenov S.A. (2017). Influence of Al_3_Ni crystallisation origin particles on hot deformation behaviour of aluminium based alloys. Philos. Mag..

[69] Nes E., Ryum N., Hunderi O. (1985). On the Zener Drag. Acta. Metall..

[70] Wang B., Wang X., Li J. (2016). Formation and Microstructure of Ultrafine-Grained Titanium Processed by Multi-Directional Forging. J. Mater. Eng. Perform..

[71] Popovic M., Verlinden B. (2013). Microstructure and mechanical properties of Al–4·4 wt-%Mg alloy (AA5182) after equal channel angular pressing. Mater. Sci. Technol..

[72] Sakai T., Jonas J.J. (2001). Plastic deformation: Role of recovery and recrystallization. Encycl. Mater. Sci. Technol..

[73] Valiev R.Z., Langdon T.G. (2006). Principles of equal-channel angular pressing as a processing tool for grain refinement. Prog. Mater. Sci..

[74] Sitdikov O., Sakai T., Goloborodko A., Miura H., Kaibyshev R. (2004). Effect of Pass Strain on Grain Refinement in 7475 Al Alloy during Hot Multidirectional Forging. Mater. Trans..

[75] Kaibyshev R., Sitdikov O., Goloborodko A., Sakai T. (2003). Grain refinement in as-cast 7475 aluminum alloy under hot deformation. Mater. Sci. Eng. A.

[76] Gazizov M., Malopheyev S., Kaibyshev R. (2014). The effect of second-phase particles on grain refinement during equal-channel angular pressing in an Al–Cu–Mg–Ag alloy. J. Mater. Sci..

[77] Buckingham R.C., Argyrakis C., Hardy M.C., Birosca S. (2016). The effect of strain distribution on microstructural developments during forging in a newly developed nickel base superalloy. Mater. Sci. Eng. A.

[78] Murashkin M.Y., Sabirov I., Medvedev A.E., Enikeev N.A., Lefebvre W., Valiev R.Z., Sauvage X. (2016). Mechanical and electrical properties of an ultra-fine grained Al–8.5 wt % RE (RE = 5.4 wt % Ce, 3.1 wt % La) alloy processed by severe plastic deformation. Mater. Des..

[79] Portnoy V.K., Rylov D.S., Levchenko V.S., Mikhaylovskaya A.V. (2013). The influence of chromium on the structure and superplasticity of Al-Mg-Mn alloys. J. Alloys Compd..

[80] Medvedev A.E., Murashkin M.Y., Enikeev N.A., Valiev R.Z., Hodgson P.D., Lapovok R. (2018). Optimization of Strength-Electrical Conductivity Properties in Al–2Fe Alloy by Severe Plastic Deformation and Heat Treatment. Adv. Eng. Mater..

